# Interactions between cancer-associated fibroblasts and T-cells: functional crosstalk with targeting and biomarker potential

**DOI:** 10.48101/ujms.v129.10710

**Published:** 2024-05-24

**Authors:** Vladan Milosevic, Arne Östman

**Affiliations:** a Department of Clinical Medicine, University of Bergen, Bergen, Norway; b Department of Oncology-Pathology, Karolinska Institutet, Stockholm, Sweden

**Keywords:** Cancer associated fibroblasts (CAF), T-cells, tumour microenvironment (TME), tumour immunity

## Abstract

Cancer-associated fibroblasts (CAFs) are a heterogeneous cell population recognized as a key component of the tumour microenvironment (TME). Cancer-associated fibroblasts are known to play an important role in maintaining and remodelling the extracellular matrix (ECM) in the tumour stroma, supporting cancer progression and inhibiting the immune system’s response against cancer cells. This review aims to summarize the immunomodulatory roles of CAFs, particularly focussing on their T-cell suppressive effects.

Cancer-associated fibroblasts have several ways by which they can affect the tumour’s immune microenvironment (TIME). For example, their interactions with macrophages and dendritic cells (DCs) create an immunosuppressive milieu that can indirectly affect T-cell anticancer immunity and enable immune evasion. In addition, a number of recent studies have confirmed CAF-mediated direct suppressive effects on T-cell anticancer capacity through ECM remodelling, promoting the expression of immune checkpoints, cytokine secretion and the release of extracellular vesicles. The consequential impact of CAFs on T-cell function is then reflected in affecting T-cell proliferation and apoptosis, migration and infiltration, differentiation and exhaustion. Emerging evidence highlights the existence of specific CAF subsets with distinct capabilities to modulate the immune landscape of TME in various cancers, suggesting the possibility of their exploitation as possible prognostic biomarkers and therapeutic targets.

## Introduction

The existence of fibroblasts as a specific cell class residing in the stromal compartment of tissues has been recognized for the first time in the 19th century by Rudolf Virchow (1). Today, fibroblasts are defined as non-epithelial, non-immune non-vascular stromal cells, responsible for the production and maintenance of the extracellular matrix (ECM) in tissues (2, 3). A century after Virchow, near the end of the 20th century, the concept of cancer-associated fibroblasts (CAFs) emerged as it has been recognized that tumour stroma could play a pivotal role in cancer biology (4). It has been thought that CAFs are a uniform cell population, characterized by the expression of vimentin, alpha smooth muscle actin (aSMA) and fibroblast activating protein (FAP) (5). Today it is an accepted truth that the stromal compartment of solid tumours contains a highly heterogeneous CAF population, the most abundant and one of the most important components of the tumour microenvironment (TME) (6–9). Many studies have been conducted over the years with efforts to classify various CAF subtypes and decode their specific functions in cancer immunity, stemness, metastasis, resistance to therapy, etc. (10–12). With this review, we aim to summarize the established knowledge about the suppressive impact CAFs have on cancer immunity with an emphasis on T-cells.

The review includes a brief summary on CAFs and innate immune respenses. This is followed by a core part on experimental studies on CAFs and T-cells, covering T-cell phenotypes regulated by CAFs, molecular mediators of CAF/T-cell interactions, specific links between certain CAF subsets and T-cell function and abundance. A summary of results relying on analyses of CAF/T-cell interactions based on analyses of human tumor samples is subsequently provided.

## Innate immune response regulated by cancer-associated fibroblasts

Tumour-associated inflammation has been recognized as an important process occurring in the TME with both pro- and anti-tumourigenic effects (13). Many studies have been conducted in recent years with the purpose of decoding a delicate interplay between CAFs and the cellular components of the immune system that either cause immune activation or immune suppression in solid tumours.

Within the cellular components of the innate immune system, macrophages are one of the cell populations that are susceptible to the modulatory influences of CAFs. In a study by Erez et al., the authors concluded that CAFs contribute to pro-tumourigenic inflammation and angiogenesis by enhancing macrophage recruitment in squamous cell carcinoma, breast cancer and pancreatic ductal adenocarcinoma (14). Selected studies on in vivo mouse models of spontaneous lymphoma and breast cancer have illustrated the exerted potential of CAFs in recruiting neutrophils, monocytes and macrophages (15, 16). In the study by Yavuz, et al., the authors have demonstrated that the presence of CAFs can recruit monocytes and affect polarization of macrophages into pro-cancerogenic M2 phenotype (17). Another in vivo study performed on breast cancer and lung metastasis murine models has shown that CAF-derived Chitinase-3 like-protein-1 (Chi3L1) stimulates macrophage migration and their conversion towards M2 phenotype; additionally, the authors have shown that CAF–derived Chi3L1 reduces CD8+ T-cell infiltration and stimulates Th2 inflammatory response (18).

Some in vitro studies have indicated that CAFs can also negatively affect natural killer (NK) cell activation. This effect has been fulfilled by CAFs secreting prostaglandin E2 (PGE2) and indoleamine 2,3-dioxygenase (IDO), which leads to inhibition of NK cell activating receptors, and by inhibition of DNAX accessory molecule-1 (DNAM-1) in hepatocellular carcinoma (19) and melanoma (20). The DC population is affected by CAFs as well. It has been shown that CAFs can negatively affect DC function by inhibiting their maturation and affecting their capability of antigen presentation (21). In addition, CAFs can induce DCs to transdifferentiate into regulatory DC (rDC), which exert impaired capability of antigen presentation, secrete inhibitory cytokines and have been also known to produce IDO, an important factor in T-cell exhaustion (21–23).

## Specific T-cell properties affected by fibroblasts

**Figure 1:**
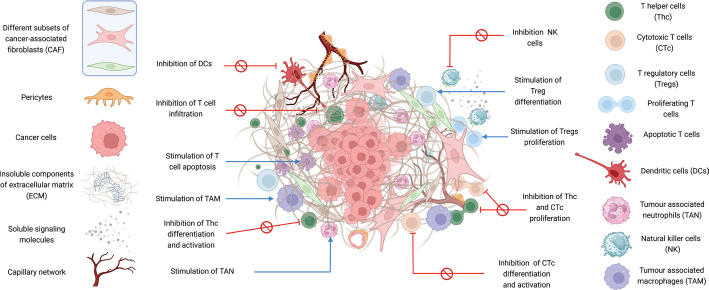
Schematic overview of the suppressing effects CAF exerts on tumour immune microenvironment. (Figure prepared using Biorender.com).

A number of in vitro and in vivo studies supported the narrative that CAFs significantly modulate adaptive immunity, particularly by altering the properties of T-cell populations. It has been demonstrated that CAFs can affect T-cell proliferation, which is crucial for mounting an effective immune response; affect T-cell migration and infiltration into the TME, a critical step for immune surveillance and tumour eradication; influence T-cell differentiation, thereby skewing the balance between effector and regulatory T-cells; and promote T-cell exhaustion, a state of dysfunction that cancer cells exploit to evade immune detection and destruction. The overview of the inhibitory effects CAFs exert on tumour immune microenvironment (TIME) has been presented in Figure 1.

### Cancer-associated fibroblasts are affecting T-cell proliferation

Several studies performed in vitro testify to the inhibitory effect CAFs exert on T-cell proliferation (reviewed in (24)). In the study by Takahashi et al. (25), the authors demonstrated that T-cell proliferation was suppressed to a higher degree by CAFs and that T-cell apoptosis was increased than what was the case with normal fibroblasts (NF) pointing towards the specific inhibitory property of CAFs. The study by Gorchs et al. (26) performed with lung cancer-derived CAFs also showed inhibited proliferation in T-cells, both when tested in coculture with T-cells and when using CAF-conditioned media (26). The effect of CAFs on T-cell proliferation has been shown to be more significant than the effect cancer cells could directly have. This has been underlined in another in vitro study performed on a pancreatic cell line, where the authors showed a higher proliferation suppression potential of CAFs than what is exerted by a cancer cell line (27). The inhibitory effect of T-cell proliferation has been shown to exist across different tumour types. For example, an *in vitro* study with human immortalized CAF has shown that CAF can suppress the proliferation of T-cells in the tumour stroma of colorectal, breast and pancreatic cancer (28).

### Cancer-associated fibroblasts are affecting T-cell migration

Multiple studies showed a negative correlation between T-cell infiltration and enrichment of the CAF component of TME with consequent higher deposition of the components of ECM. Ex-vivo models of lung cancer further helped in understanding the role of CAFs in T-cell exclusion. Namely, the study proved that high interstitial fluid pressure and tight network of ECM deposited by CAFs in lung cancer stroma affects antitumour immunity by physically impairing migration and positioning of T-cells within the tumour tissue (29). In the same study, the authors concluded that fibronectin-rich regions, mostly surrounding tumour islands, inhibit T-cell motility. The exclusion of T-cells from the TME can carry significant clinical implications, as it may impact the practical application and effectiveness of immunotherapies. For example, in the study by Ford et al., the authors concluded that CAF-rich tumours show poor response to anti-PD-1/PD-L1 immunotherapy (IT) and that this therapy failure is caused by the exclusion of CD8+ T-cells from the tumour stroma (30).

In addition to creating mechanical barriers for T-cell migration and infiltration of T-cells, there is an increased amount of evidence that CAFs utilize signalling molecules that actively affect the motility and infiltration potential of T-cells. For example, it was observed that CAFs residing in the TME secrete molecules that cause the exclusion of CD8+ T lymphocytes from the tumour islands (31). In another study performed on oesophageal cancers, the authors showed that in CAF-rich tumours, infiltration of CD8+ T-cells is limited to the peritumoural area and that intratumourally infiltration is significantly reduced due to the higher levels of CAF-secreted IL-6 (32).

### Cancer-associated fibroblasts are affecting T-cell differentiation and exhaustion

It has been known that fibroblasts residing in lymph nodes (fibroblastic reticular cells, FRC) use chemokines to control the physiological functions of T lymphocytes in healthy lymph nodes (33). Building on this knowledge, a number of studies have indicated that CAFs can utilize this immunomodulatory potential to influence T-cell differentiation and exhaustion in the TME. For example, in the study by Gorchs et al., the authors presented convincing results on CAFs being involved in stimulating FoxP3 and immune checkpoints (T-cell immunoglobulin domain and mucin domain 3 (TIM-3), programmed cell death protein 1 (PD-1), lymphocyte activation gene 3 (LAG-3) and cytotoxic T lymphocyte-associated protein 4 (CTLA-4) expression on CD4+ T-cells, as well as inhibition of T-cell immunoreceptor with immunoglobulin and ITIM domain (TIGIT) expression on CD8+ cells and HLA-DR expression on both CD4 and CD8 proliferating T-cells (27), indicating a strong immunomodulatory effect of CAFs.

Moreover, It has been known that specific CAF subsets cause attraction and support to the T regulatory (Tregs) cell population within TME (34). An in vivo study by Huang et al. demonstrated that CAFs can directly induce the transformation of T helper (Th) cells into Tregs in pancreatic cancer (35).

Adversely, several studies suggest that CAFs can affect cytotoxic T lymphocyte (CTL) activation in TME. In the study by Ersek et al., the authors studied the effect of melanoma-derived CAFs on CTL. They concluded that CAFs were responsible for interfering with CD8+ cell activation by interfering with cell signalling, reducing levels of granzyme B and activation marker CD69. Additionally, the authors showed that melanoma-derived CAFs were causing upregulation of immune checkpoints TIGIT and B- and T lymphocyte attenuator (BTLA) (31).

Multiple studies described the indirect effect of CAFs on T-cells. In the study by de Monte et al., the authors suggested that CAFs can skew T-cell activation towards T helper type 2 (TH2) indirectly by affecting the DCs population (36). Cancer-associated fibroblast-controlled DC has been also known to affect CD8 T-cell differentiation in oesophageal squamous cell carcinoma and colorectal cancer (37).

Dendritic cells are not the only immune cells CAFs use to affect T-cell function indirectly. In triple-negative breast cancer, CAF-secreted C-X-C motif chemokine 16 (CXCL16) has been shown to attract monocytes in the tumour site (38). It also has been known that CAFs exert their immunosuppressive effect by affecting the macrophage population (39). Namely, CAFs affect macrophages to undergo M2 polarization and M2 attraction and retention in TME. M2 macrophages then by expressing PD-1 inhibitory molecule and by secreting TGF-β, arginase and IL-10 affect the proliferation of T-cells and function of CD8 + T-cells (40).

## Molecular mechanisms behind cancer-associated fibroblasts-controlled immunosuppressive properties

### Cancer-associated fibroblasts mechanisms affecting T-cell proliferation

**Figure 2:**
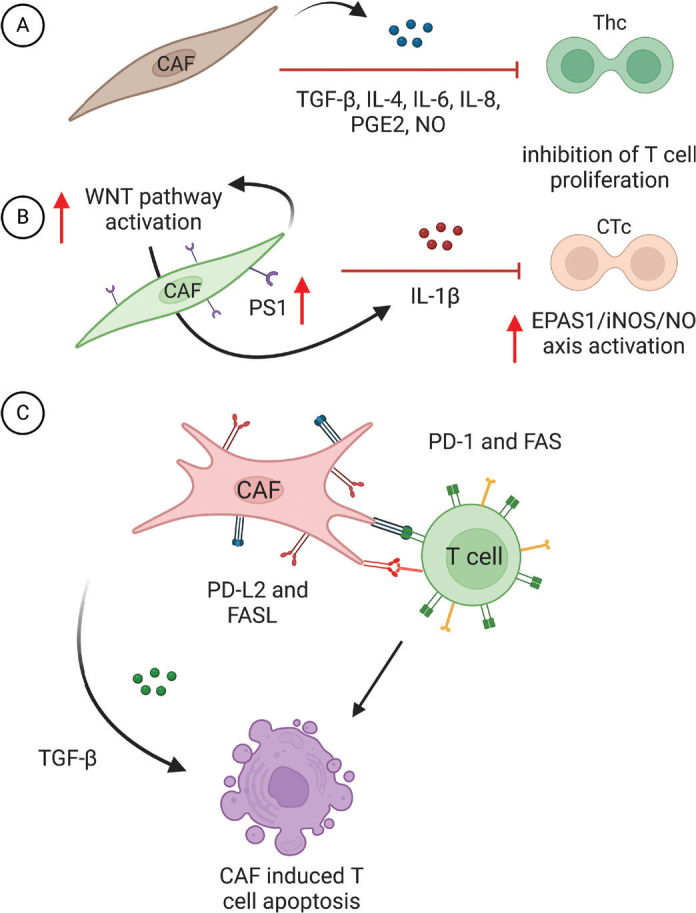
The molecular mechanisms of CAF-induced T-cell apoptosis and suppression of T-cell proliferation. (A) Cancer-associated fibroblasts inhibit T-cell proliferation through the secretion of signalling molecules. (B) Increased expression of Presenilin-1 (PS1) in CAFs activates the WNT pathway, leading to the release of IL-1β. This consequently causes activation of EPAS1/iNOs/NO signalling cascade in cytotoxic T-cells, inhibiting their proliferation. (C) DirecT-cell-cell contact between CAFs and T-cells, mediated by PD-L2 and FASL on the surface of CAFs, as well as PD-1 and FAS on T-cells, induces apoptosis in T-cells. (Figure prepared using Biorender.com)

In the study by Gorchs et al. performed on lung cancer-derived CAFs, the authors indicated that the proliferation inhibitory potential of CAFs could come from the molecules secreted by CAFs that can affect T-cells in the paracrine manner (26). By analysing the CAF secretome they identified immunomodulatory molecules such as PGE2, IL-4 and TGF-β that could have immunosuppressive potential and could likely be responsible for suppressing proliferation in T-cells in their setting (Figure 2A) (26).

Some studies indicated that knocking down the upstream CAF regulator *AKT3* consequently causes lower expression of TGF-β, PD-L1, PD-L2, IL-6 and IL-8, which consequently increased T-cell proliferation in the experimental setting, indicating its role in the CAF-controlled immune regulation (41). In addition to this, PD-L2 and fatty acid synthase ligand (FASL) production by CAFs has been shown to induce a highly targeted apoptotic process in cytotoxic T-cells by interaction with PD-1 and FAS on their surface (Figure 2C) (42). In the study by Zhang et al., the authors indicated preselin 1 (PS1) as another upstream regulator in CAFs responsible for controlling the inhibiting effect on T-cell proliferation through activation of the WNT/β-catenin pathway (43) possibly through its stimulation of IL-1β secretion (43, 44), which has been known to promote immune suppression and exclusion (45) (Figure 2B). Another mechanism of CAF-controlled inhibition of T-cell proliferation could be CAF-secreted NO (46), mimicking FRC-controlled proliferation of T-cells in healthy lymph nodes (33, 46).

Also, Mei et al. suggested that the toll-like receptor 4 (TLR4)-dependent pathway in CAFs could have an immunosuppressive effect and a negative effect on T-cell proliferation, that can be reversed using the active compound found in cinnamon, cinnamaldehyde (47).

### Cancer-associated fibroblasts mechanisms affecting T-cell migration

**Figure 3:**
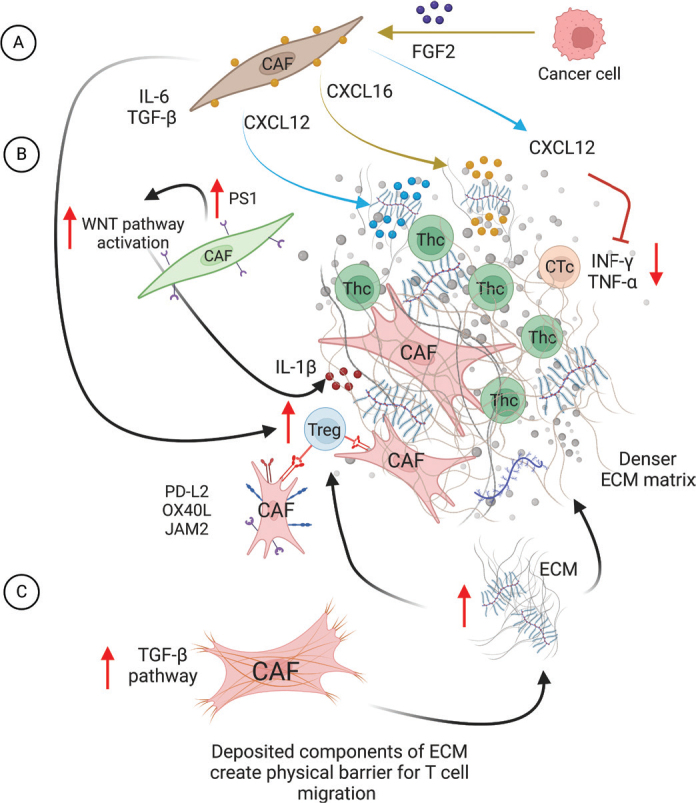
Cancer-associated fibroblasts affect T-cell migration and infiltration. (A) Under the influence of FGF2 secreted by cancer cells, CAFs secrete CXCL16, which together with CXCL12 has a high affinity towards proteoglycans in ECM, and causes imprisonment of T-cells in the ECM not allowing tumour islet infiltration. Cancer-associated fibroblasts secreted CXCL12 blocks INF-γ and TNF-α production by T-cells, impairing their motility and function. (B) Cancer-associated fibroblasts can specifically support migration and infiltration of Tregs through secretion of IL-6, TGF-β and IL-1β. Additionally, CAFs attract and mobilize Tregs through direct cell-cell contact mediated by surface molecules PD-L2, OX40L and JAM2. (C) Through increased production of insoluble components of ECM mediated by hyperactivity of the TGF-β pathway in CAFs, they create a denser ECM matrix that acts as a physical barrier discriminately towards Thc and CTc, allowing survival and infiltration of Tregs. (Figure prepared using Biorender.com)

The main mechanism in CAF-governed T-cell exclusion is building physical barriers in the ECM although there are more complex molecular mechanisms involved as well. Chen et al., using *in vivo* mice models of lung cancer and melanoma, demonstrated that whole cell vaccine modified to express FAP showed a directed effect both towards tumour cells and FAP-expressing CAFs. This consequently led to lower expression of both FAP and collagen I, and increased infiltration of CD8+ T-cells (48). This effect is shown to be significantly stronger when compared to the effects of non-FAP modified whole cell tumour vaccine indicating stronger, double-sided, potentially therapeutic effects in removing FAP-expressing CAFs (48). The study by Gorchs et al. indicated the effect of CAFs on T-cell migration, by CAFs secreting CXCL12 (26) as well as CAFs causing immunosuppressive effects by inhibition of INF-γ and TNF-α production by T-cells (Figure 3A). Furthermore, It has been confirmed that the production of C-X-C motif chemokine 12 (CXCL12) is dependent on CAF activation by fibroblast growth factor beta (FGF2), secreted by cancer cells and is associated with CD8+ T-cell exclusion in cancer (49). Another chemokine produced by CAFs and caused by stimulation of CAFs by secretion of TGF-β by cancer cells is CXCL16 (49). It exists in a soluble and a transmembrane form and it can act both as an adhesion molecule and gradient chemoattractant (similar to CXCL12) (49, 50) (Figure 3A).

In the study by Zhang et al., the authors performed the analysis of genes differentially expressed in CAFs derived from ovarian cancer comparing the expression pattern with normal fibroblasts (43) where they identified genes associated with T-cell infiltration. One of the highly expressed genes *PS1* was demonstrated to play a crucial role in T-cell exclusion through WNT/β-catenin pathway-directed CAF activation and aSMA and FAP expression (43) (Figure 3B).

T-cell exclusion, as demonstrated in the study by Mariathasan et al. on the murine model of urothelial carcinoma (51) and confirmed by Desbois et al. on ovarian cancer, could be caused by higher activity of the TGF-β pathway in CAFs due to the increased production of the components of ECM creating a physical barrier for T-cell migration (52) (Figure 3C).

CD8+ T-cell exclusion has been known to be caused by CAFs secreting signalling molecules such as IL-6 and TGF-β (53). The effect of the T-cell exclusion seems to be biased specifically towards CD8+ T-cells, and it favours CD4+ T-cells, especially the Treg population (24, 54) (Figure 3A). The mechanism behind this may be related to the differential cytotoxic effect that a high-stiffness ECM could have on different T-cell populations (54) (Figure 3C).

### Cancer-associated fibroblast mechanisms affecting T-cell differentiation and exhaustion

**Figure 4:**
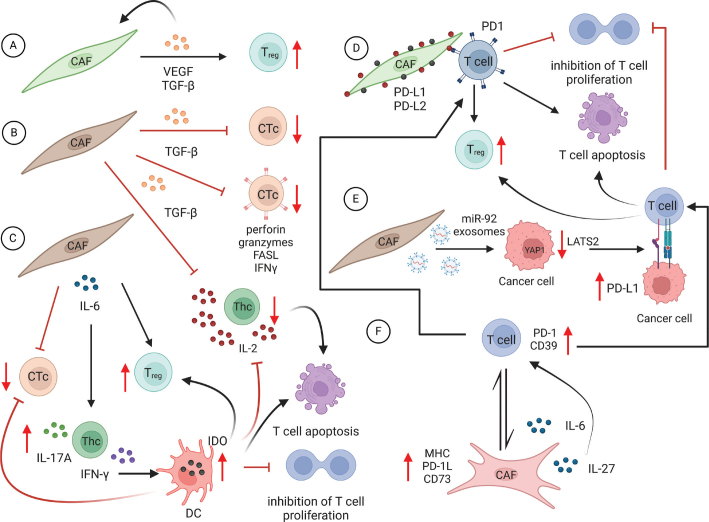
The molecular mechanism of CAFs affecting T-cell differentiation and exhaustion. (A) Cancer-associated fibroblasts secrete auto-stimulating molecules VEGF and TGF-β, which cause differentiation of Tregs. (B) Cancer-associated fibroblasts-secreted TGF-β also impairs the differentiation of CTc and affects the production of CTc functional molecules such as perforin, granzymes, FASL and INFγ. In addition, TGF-β inhibits Thc and their release of IL-2. (C) IL-6 secreted by CAFs has an inhibitory effect on CTc differentiation and it stimulates the differentiation of Tregs. In addition, it stimulates Thc production of INF-γ and IL-17A. Although this activation still ends up having an inhibitory effect, through INF-γ inducing IDO expression in DC, consequently causing T-cell apoptosis, and inhibiting IL-2 release and T-cell proliferation. (D) Through the direct cell-cell contact mediated by PD-L1 and PD-L2 on CAFs and PD-1 on T cells, CAFs cause Treg differentiation, inhibition of T-cell proliferation and T-cell apoptosis. (E) Through miR-92 containing exosomes, CAFs induce YAP1 nuclear translocation in cancer cells that reduces their expression of LATS2. In return, this causes higher expression of PD-L1 on the surface of cancer cells, which in contact with PD-1 on T-cells causes the same effect as in D. (F) Bidirectional interaction between CAFs and T-cells mediated through causes the higher expression of PD-1 and CD39 on T-cells, inducing the same endpoint effects as in E and D. (Figure prepared using Biorender.com)

In the study on lung adenocarcinoma, Kinoshita et al.’s in vitro experiments suggested that CAFs isolated from tumours with a high Treg count were capable of inducing Treg differentiation from healthy donor PBMCs, potentially through the secretion of TGF-β and VEGF (55) (Figure 4A). Cancer-associated fibroblast-secreted TGF-β has been also indicated as a possible cause of suppressing cytotoxic T cell differentiation into T effector memory cells, inhibiting their proliferation and promoting their apoptosis (56). In another study, performed on a murine model of pancreatic cancer, the authors showed that inhibiting TGF-β increased the amount of naïve Tregs and reduced the number of effector and memory Tregs. In addition to this, the authors concluded that TGF-β inhibition suppressed Tregs-controlled CD8 + T-cell suppression (57) (Figure 4B).

Activation of the TGF-β pathway in CAFs is also held responsible for the suppression of anti-cancer immunity and higher occurrence of Tregs and M2 macrophage populations, and it has been associated with the expression of podoplanin (PDPN) in CAFs in non-small cell lung cancer (58). Furthermore, the study by Desbois et al. confirmed the role of TGF-β signalling in suppressing the activity of CD8+ T-cells (52). TGF-β production by CAFs has been associated with the suppression of CD8+ T-cell activity by inhibiting expression of the key cytotoxic genes responsible for the synthesis of perforin, granzyme A and B, FAS ligand and IFN-γ (Figure 4B) (59). This effect is achieved by the activation of SMAD and ATF1 transcription factors, directly and selectively stopping the transcription of the cytotoxic genes in CD8+ T-cells (59), as well as affecting the production of IL-2 by T-cells (60). It has been known that CAFs are a significant source of IL-6 production in the tumour stroma (61, 62). In physiological conditions, IL-6 is being expressed by FRC with the aim of controlling T-cell differentiation in Lymph nodes (33, 63) indicating a similar effect on T-cells in TME (24). High IL-6 production has been also known to push T-cells towards immunosuppressive phenotypes promoting FoxP3 differentiation and CD8+ T-cell suppression (32). This effect of IL-6 has been confirmed in the study by Tsukamoto et al. on the murine melanoma model (64), where the authors reported that targeting IL-6 signalling in addition to anti-PD-1-PD-L1 blockage synergistically enhanced tumour-specific Th1 response (64). Another study discusses the induced IFN-γ and IL-17A production in T-cells through IL-6 secretion seemingly stimulating the T-cell activity in cancer (65). Although IFN-γ is a well-known activator of CD8+ T lymphocytes, it has been known that it also induces IDO synthesis by mesenchymal stem cells and DCs, having an immunosuppressive effect by promoting Treg differentiation, inhibiting T-cell proliferation, inhibiting CTc function and Thc function, reducing IL-2 release by Thc and promoting apoptosis in T-cells (Figure 4C) (66–72), indicating a similar mechanism of immunosuppression in the TME.

It has been shown that CAFs compared to NF express higher levels of coregulatory molecules (PD-L1 and PD-L2) and cytokine genes (*IL6, CXCL8, TNF, TGFB1 and VEGFA*), including genes associated with leukocytic extravasation and paxillin signalling pathway (paxillin plays an important role in assembly/disassembly of focal cell adhesions) indicating the potential of CAFs in shaping immunosuppressive landscape of TME using different strategies including recruitment and support of immunosuppressive cells of both adaptive and innate immunity (25).

It has been known that CAFs can affect T-cell response by secreting WNT2 which then causes activation of the WNT/β-catenin pathway in DCs that cause suppression of the T-cells by affecting the SOCS3/p-JAK2/p-STAT3 signalling pathway (37).

Other studies suggested more direct communication between CAFs and T-cells that cause T-cell suppression. For example, in the study by Takahashi et al., the authors demonstrated the inhibitory effect of CAFs that is exerted through the expression of inhibitory checkpoint ligands PD-L1 and PD-L2 on CAF surface that in contact with T-cells exert an inhibitory effect (25) (Figure 4D). Furthermore, the authors comment on the similar effect caused by CAF-secreted auto-stimulating molecules such as VEGF and TGF-β (25). A recent study by Hu et al. (73) suggested the possibility that CAF – T-cell direct interaction (potentially mediated by the PD-1/PD-L1 pathway) may contribute to the differentiation of Tregs (Figure 4D).

In the study by Dou et al., the authors concluded that CAFs can create immunosuppressive TME indirectly by promoting miR-92/PD-L1 pathway activity in cancer cells through shedding miR-92-containing exosomes. These exosomes deliver miR-92 to the cancer cells consequently causing the suppression of large tumour suppressor kinase 2 (LATS2) and nuclear translocation of yes1 associated transcriptional regulator (YAP1) and subsequent upregulation of PD-L1. Elevated levels of PD-L1 cause suppression of T-cells’ and NK cells’ function against cancer cells (74) (Figure 4E).

In the study by Chauhan et al., the authors observed a bidirectional interaction between activated T-cells and CAFs. Activated T-cells induce CAFs to upregulate major histocompatibility complex (MHC) molecules, PD-1 ligands and CD73, and increase the secretion of both IL-6 and IL-27. In turn, CAFs promote the expression of co-inhibitory molecules and the ectonucleotidase CD39 on T-cells, contributing to an exhausted T-cell phenotype (75–77) (Figure 4F). Additionally, IL-27 has been also known to trigger T-cell exhaustion by inducing the expression of TIM-3 and IL-10 secretion via the transcription factor NFIL3 (nuclear factor interleukin-3-regulated protein), which leads to diminished and dysfunctional T-cell effector function (78). Notably, IL-27 has been known for decades as a pro-immune cytokine, supporting the activation of CTLs by inducing their proliferation and production of granzyme B (79), indicating potential dual roles in immune regulation.

One of the mechanisms by which CAFs can cause impairment and anergy of CD8+ T-cells is through their elevated arginase activity. In the study by Ersek et al, the authors concluded that CAFs via elevated arginase activity, and secretion of soluble factors (CXCL12), caused dysregulation of extracellular signal-regulated protein kinases 1 and 2 (ERK1-2) and nuclear factor kappa B (NF-kB) signalling by the reduced levels of L-arginine in CD8+ T-cells. Cancer-associated fibroblasts also exerted upregulation of V-domain Ig suppressor of T-cell activation (VISTA) and herpes virus entry mediator (HVEM) (which serve as BTLA ligands) further contributing to CD8+ cells anergy and inhibition (31).

Cancer-associated fibroblasts can impair T-cell activity indirectly, by affecting the activity of other immune cells (55). It has been known that CAFs can affect DC cell differentiation and maturation, inducing the inhibitory CD11c + DC phenotypes, which due to secretion of WNT2, and consequent activation of WNT/β-catenin pathway in CD8+ T-cells, cause inhibition of CD8+ T-cell priming (37).

## Specific cancer-associated fibroblasts subsets responsible for controlling immunosuppressive tumour microenvironment

The definition of novel CAF subsets is a highly active research area. Multiple methods have been used including FACS analyses of tumour single-cell suspension (80), single-cell RNA seq (81–83) or multiplex staining of tissue (84). Emerging findings suggest that these subsets indeed differ with regard to their immune modulatory capacity.

The immunosuppressive property of CAF-S1 has been confirmed in breast cancer by Costa et al., wherein in vitro conditions CAF-S1 cells showed higher attractive capability towards Treg cell phenotypes, through secretion of CXCL12 and that they are capable of direct interaction with Tregs, retaining them on their surface using specific membrane proteins (OX40 ligand (OX40L), PD-L2 or junctional adhesion molecule (JAM2) (80). CAF-S1 subset has been known to control the production of the CXCL12β chemokine isoform through miR-141/200a post-transcriptional regulation, which is crucial for attracting regulatory T-cells (85). In addition to this, CAF-S1 was shown to have a higher potential in differentiation and activation of Tregs, through CD276 (B7H3), CD73 (NT5E) and dipeptidyl peptidase 4 (DPP4), and enhanced Treg cells capability of suppressing effector T-cell proliferation (80). Dipeptidyl peptidase 4 (DPP4) is, in fact, a FAP dimerization co-molecule, known to cleave the chemoattractant C-X-C motif chemokine 10 (CXCL10) responsible for attracting effector T-cells (80, 86, 87). A more recent study by Kieffer et al. defined two FAP expressing CAF subsets, originating from the CAF-S1 phenotype, named ecm-myCAF and TGF-β-myCAF that were shown to be significantly associated with immunosuppressive TME. Namely, myCAF and TGF-β-myCAF rich tumours showed a high frequency of PD-1+, CTLA-4+ and TIGIT+ CD4+ T lymphocytes and a significantly lower number of CD8+ T lymphocytes (34). More specifically, the authors concluded that the ecm-myCAFs stimulated the expression of PD-1 and CTLA-4 on the surface of Tregs, which consequently stimulated the expansion of TGF-β-myCAFs creating a positive feedback loop enhancing the immunosuppressive environment (34).

Fibroblast activating protein and aSMA-expressing CAFs under the influence of cancer cells are known to secrete CXCL16 which is a powerful chemoattractant associated with T-cell exclusion (50). Another property by which FAP could cause an increase in the amount of Tregs in TME is due to stimulation of their proliferation. Numerous other studies have indicated the involvement of FAP+ CAFs in augmenting the Treg infiltration in tumour TME and consequent immunosuppression. In the study by Coto-Llerena et al. on colorectal cancer, the authors concluded that FAP stroma expressing tumours showed higher infiltration of macrophages and monocytes and high number of Tregs, with opposed low levels of NK cells and T helper 1 cells, contributing to poor prognosis (88).

It has been known that FAP-positive CAFs secrete stromal cell-derived factor 1 (SDF-1, also known as CXCL12), a potent chemokine (89) that has an affinity to bind to the components of ECM (e.g. heparan sulfate proteoglycans, (90)) creating a concentration gradient capable of attracting and leading CD8+ T-cells by binding to their receptor CXCR4 (C-X-C chemokine receptor type 4). This causes strong attraction of CD8+ T-cells to the stromal region and consequent imprisonment and exclusion from the tumour islets limiting CD8+ T-cells migration and infiltration capabilities (91–93). There have been efforts made to exploit this type of CAF-T-cell interactions for the development of new therapies, where CXCR4 inhibitions showed promise in the therapy of pancreatic ductal adenocarcinoma (94). Another chemokine associated with T-cell exclusion is CXCL16, known to be secreted by CAFs expressing FAP and aSMA (49).

Production of IL-6 was associated with FAP expressing/aSMA non-expressing (or aSMAlow) CAF phenotypes (CAF-S1 based on the classification by Costa et al. (80), or inflammatory CAF phenotype, based on classification by Öhlund et al. (95). Adversely, in the study by Kato et al., the authors demonstrated that aSMA+ CAF secrete IL-6 which is a consequent cause of decreasing the number of CD8+ T-cells and an increase in the number of Tregs in the TME (32).

In the study performed on pancreatic cancers, Gorschs et al. reported that a high-density matrix, localized in the areas surrounding tumour islands, is characterized by high numbers of aSMA-expressing CAFs (27). In addition, the authors associated high expression of aSMA in CAFs with higher expression of immune checkpoints on T-cells and demonstrated that aSMA + CAFs strongly inhibited T-cell proliferation in a contact-independent manner by secretion of prostaglandin E2 (27). Alpha smooth muscle actin + CAFs have been known to impend T-cell activation and cause T-cell exhaustion through fibroblast growth factor receptor (FGFR) signalling. In the study by Chen et al., the authors concluded that fibroblast growth factor 2 (FGF2) secreted by CAFs leads to the activation of the FGFR signalling pathway in T-cells, causing upregulation of protein sprouty homolog 1 (SPRY1). Protein sprouty homolog 1 inhibits the activity of NF-kB, nuclear factor of activated T-cells (NFAT) and Ras MAPK signalling pathways in T-cells, and consequently lowers the production of IFN-γ, TNF-α and granzyme B in CD8 + cells impairing their cytotoxic function (96). FGFR2+ CAFs have been known to cause an immunosuppressive environment indirectly by affecting DC through WNT/β-catenin pathway activation (37). Takahashi et al. pointed out in their study on CAFs coming from head and neck squamous cell carcinoma that *AKT3* is a key player in reprogramming CAFs into myofibroblast phenotype with demonstrated immunosuppressive properties (41).

The specific phenotype of CAFs that has been associated with T-cell proliferation inhibition through NO synthesis and secretion in breast cancer was FAP/PDPN-expressing CAFs (46). iCAF phenotype is known to exert upregulation of inflammatory pathways such as TNF-α, IL-2/STAT5, complement pathway and IFN-γ (81). Podoplanin-positive CAFs are also known to be associated with a higher occurrence of Tregs and M2 macrophage populations in non-small cell lung cancer (58).

Single-cell RNAseq in iCAF phenotype showed higher expression of genes associated with the production of ECM proteins, notably upregulation of genes *HAS1 and HAS2* responsible for the production of hyaluronan (81), as well as *AGTR1,* known for its function in stimulating the production of collagen and fibronectin (81, 97).

## Supportive data from analyses of clinical samples

As summarized above, a large set of pre-clinical and model studies suggest functional and specific interactions between CAF subsets and various types of immune cells. Following up on these studies, a series of analyses of clinical samples have now been carried out to consolidate these model-based studies regarding clinical relevance.

This summary follows a structure that first discusses selected evidence for positive and negative associations between CAF subsets and immune cells regarding abundance or density. This is followed by a section on findings describing T-cell/CAF subset spatial enrichments, and other evidence for CAF-mediated regulation of T-cell exhaustion. Finally, a few examples are given where CAF status has been linked to response to immune therapies.

Based on the present data situation, most examples are derived from analyses of breast, lung and pancreas cancer.

### Abundance associations

Already the original study on the S1-S4 breast cancer CAF subsets detected associations between abundance of CAF subsets and T cell subsets. Based on IHC analyses of more than 250 breast cancers, a positive association was detected between the S1 subset and FoxP3+ cells in the triple-negative group. This association was not seen regarding S1 abundance and total T cells (CD3+) or CD8 positive cells (80). A follow-up study that focussed on single-cell RNAseq-defined subgroups of the S1 group refined these analyses, in studies using either FACS-determined T-cell composition and analyses of larger data sets relying on novel gene signatures. This study indicated that the FoxP3 association was linked to ‘ecm-myCAF’ and ‘TGF-β-myCAF’, and could also show associations of these subsets and CD4+ T lymphocytes expressing high levels of immune checkpoints, including PD-1 and CTLA-4 (34). Conversely ‘detox-iCAF’ and ‘IL-iCAF’ were correlated with an immunocompetent environment (Kieffer *34*). An additional study from the same group has also linked the FoxP3-association to a CD73+ subset of S1 cells (98).

Also, lung cancer analyses have uncovered significant associations between CAF subsets and the abundance of immune cells. A multiplex staining study based on analyses of two cohorts, each with more than 300 cases, classifying fibroblasts into 15 sub-groups based on FAP, PDGFRA (platelet-derived growth factor receptor alpha), PDGFRB (platelet-derived growth factor receptor beta) and aSMA showed that the poor prognosis-associated FAP+/PDGFRA-/ PDGFRB+/aSMA+ was associated with CD163+ cells (84) (Pellinen, JNCI). Furthermore, another study classifying lung CAFs based on FAP, aSMA, myosin heavy chain 11 (MYH11) and alcohol dehydrogenase 1B (class I), beta polypeptide (ADH1B) noted positive associations between FAP+ CAF and the enrichment of inflammatory secreted phosphoprotein 1 (SPP1)+ monocyte-derived macrophages and IgG+ plasma cells (99) (Grout, Can Disc).

## Associations between cancer-associated fibroblasts subsets and T-cell activity or localization

The overall concept that CAF subsets affect immune cell activity predicts that CAF composition of tumours should not only be correlated with the abundance of various immune cells but also with their localization and activity states. Recent studies have provided preliminary support for this notion.

Evidence for specific spatial enrichment between CAF and T cell subsets has been obtained in spatial transcriptomics analyses of breast cancer where ‘iCAF’ and ‘myCAF’ subsets showed inverse spatial associations in relationships to CD4 and B-cells, with ‘iCAF’ showing positive spatial enrichment (100).

Associations between particular CAF subsets and T-cell exhaustion have been described in different tumour types. Fibroblast subsets linked to T-cell exhaustion include the ecm-myCAF and TGF-β-myCAF subsets in breast cancer and a FAP+/PDGFRA- subset in lung cancer (34; 84). Similarly, findings on positive associations between FAP+ CAFs and exhaustion markers have also been made in another lung cancer study (99). These findings have been extended in spatial transcriptomics studies which have identified co-localization of certain CAF subsets and exhausted T cells in head and neck cancer (101).

Furthermore, observations in different tumour types have also demonstrated associations between CAF status and infiltration of T cells into tumour nests supporting the idea that specific CAF subsets can create a local environment surrounding tumour nests that favours or prevents T cell infiltration. Key findings in this context are analyses in lung cancer which showed that MYH11/aSMA+ and FAP+/aSMA+ and fibroblasts were associated with lower tumour nest infiltration of T cells, whereas no such associations were seen for the ADH1B+ or single FAP+ subsets (99). Similar findings were recently reported in an IMC-based lung cancer study, where ‘mCAFs’ (FAPhigh) were associated with the exclusion of T cells from tumor cell nests. (102).

### Associations between cancer-associated fibroblasts subset composition and response to immunotherapy

The findings above collectively suggest that CAF subsets have the potential to be developed into response-predictive markers for immune therapies through their ability to modulate T cell abundance, localization and activity states. Some early studies in this area have been presented in recent years.

These studies, on the one hand, provide early support for this notion, but there are also common limitations of studies. Outcome analyses have been performed on IT-treated cases only, limiting the possibility of separate effects of CAFs on overall tumour aggressiveness and the efficacy of IT. Also, the CAF status of clinical samples is most commonly deduced using various signatures and deconvolution approaches, rather than relying on more robust high-content in situ profiling with multiplex antibody staining or spatial transcriptomics. Furthermore, many studies restrict outcome associations to gene-set-enrichment analyses where responder/non-responder signatures are compared with CAF signatures, instead of relying on standard outcome analyses such as Log Rank-tests or Cox-regression analyses. Nevertheless, a series of promising findings have been made.

Fibroblast subsets of ‘myCAF/TGF-β-activated’ type have been linked to poor effects of immune therapy in a number of tumour types including bladder cancer, pancreas cancer, kidney cancer, lung cancer and melanoma (34, 82, 103). Outcome associations have been shown as Log-Rank/Kaplan-Meier analyses (e.g. 82, 103) or as differences between responders and non-responders in signature scores (34). Not only CAF abundance has been linked to IT sensitivity but also more complex metrics. A liver cancer study provided early indications that the spatial connections or niche preferences of CAFs differed between liver cancer cases responding and not responding to IT (104).

## Future perspectives

The dynamic and expansive research area of CAF/T-cell interactions suggest optimism, and also points towards a series of research directions to be further developed.

Regarding mechanistic studies it seems productive to continue with efforts where candidate pathways are subjected to stringent analyses using multiple and relevant mouse models to investigate combination therapy approaches where perturbations of CAF/T-cell interactions are combined with use of different checkpoint inhibitors.

Tissue profiling of clinical samples are also likely to continue to be productive steps towards more direct translational efforts. Importantly, present studies are largely restricted to primary tumors and analyses of metastases are therefore highly warranted.

From the perspective of the potential of CAF/T-cells interactions as predictive markers for immune therapy, future studies relying on trial-derived material with control and immune treated patients should be high priority.

Together such studies should hopefully continue to yield findings that will maintain and increase interest and funding in this area. Ultimately, such future efforts, with academy and industry interacting, will hopefully bring forward novel biomarkers of clinical utility and new therapies.
